# Potential synergy between PSMA uptake and tumour blood flow for prediction of human prostate cancer aggressiveness

**DOI:** 10.1186/s13550-021-00757-y

**Published:** 2021-02-09

**Authors:** Mads Ryø Jochumsen, Jens Sörensen, Lars Poulsen Tolbod, Bodil Ginnerup Pedersen, Jørgen Frøkiær, Michael Borre, Kirsten Bouchelouche

**Affiliations:** 1 Department of Nuclear Medicine & PET-Centre, Aarhus University Hospital, Palle Juul-Jensens Boulevard 165, 8200 Aarhus N, Denmark; 2 Department of Clinical Medicine, Aarhus University, Aarhus, Denmark; 3 Department of Radiology, Viborg Regional Hospital, Viborg, Denmark; 4 Department of Surgical Sciences, Uppsala University, Uppsala, Sweden; 5 Department of Radiology, Aarhus University Hospital, Aarhus, Denmark; 6 Department of Urology, Aarhus University Hospital, Aarhus, Denmark

**Keywords:** Prostate cancer, PSMA, Tumour blood flow, Rubidium, ISUP grade group

## Abstract

**Background:**

Both prostate-specific membrane antigen (PSMA) uptake and tumour blood flow (TBF) correlate with International Society of Urological Pathology (ISUP) Grade Group (GG) and hence prostate cancer (PCa) aggressiveness. The aim of the present study was to evaluate the potential synergistic benefit of combining the two physiologic parameters for separating significant PCa from insignificant findings.

**Methods:**

From previous studies of [^82^Rb]Rb positron emission tomography (PET) TBF in PCa, the 43 patients that underwent clinical [^68^Ga]Ga-PSMA-11 PET were selected for this retrospective study. Tumours were delineated on [^68^Ga]Ga-PSMA-11 PET or magnetic resonance imaging. ISUP GG was recorded from 52 lesions.

**Results:**

[^68^Ga]Ga-PSMA-11 maximum standardized uptake value (SUVmax) and [^82^Rb]Rb SUVmax correlated moderately with ISUP GG (rho = 0.59 and rho = 0.56, both p < 0.001) and with each other (r = 0.65, p < 0.001). A combined model of [^68^Ga]Ga-PSMA-11 and [^82^Rb]Rb SUVmax separated ISUP GG > 2 from ISUP GG 1–2 and benign with an area-under-the-curve of 0.85, 96% sensitivity, 74% specificity, and 95% negative predictive value. The combined model performed significantly better than either tracer alone did (p < 0.001), primarily by reducing false negatives from five or six to one (p ≤ 0.025).

**Conclusion:**

PSMA uptake and TBF provide complementary information about tumour aggressiveness. We suggest that a combined analysis of PSMA uptake and TBF could significantly improve the negative predictive value and allow non-invasive separation of significant from insignificant PCa.

## Background

Prostate cancer (PCa) is a heterogeneous disease, and consequently, an important challenge in PCa management is differentiation of clinically significant PCa from insignificant PCa that will not cause symptoms or affect the patient’s lifetime [1]. Multiple potential predictive and prognostic biomarkers have been evaluated in studies for assessment of the tumour’s biological potential, including genetic profiling, and various imaging modalities [1–3].

Prostate-specific membrane antigen (PSMA) is a transmembrane protein, which is upregulated in most PCa. PSMA tracers are indisputably excellent for detection of PCa, both for detection of local recurrence and for staging of primary tumour, bone-, and lymph node metastases [4, 5]. The uptake of PSMA in the primary tumour have also been proposed as a marker of PCa aggressiveness in a number of studies [6–9]; however, in a recent large study on high-risk patients, we have demonstrated a large overlap in tumour PSMA uptake between International Society of Urological Pathology (ISUP) Grade Groups (GG) [10].

The PSMA protein is an enzyme called glutamate carboxypeptidase, which splices polygammaglutamate-folate into folate and glutamate. In the small intestine, it is referred to as folate hydrolase, which enables freeing of folate that can be absorbed from the diet. In nervous tissue it is often referred to as N-acetyl-L-aspartyl-L-glutamate peptidase, which increases the concentration of the excitatory neurotransmitter glutamate in the extracellular space, and was studied as a potential new drug target in treating various neurological and psychiatric diseases [11]. Two studies by Yao et al. showed that PSMA expression was associated with a higher cellular folate uptake [12, 13]. This could provide a rationale behind a correlation between PSMA expression and PCa aggressiveness, as folate is necessary for DNA synthesis and cell division, and hence, a higher accessibility may give a proliferative advantage. On the other hand, Ghosh et al. found that PSMA expression actually reduced the invasiveness of PCa cell lines and that a knock down of PSMA expression increased invasiveness fivefold [14]. Whether the enhanced availability of the glutamate neurotransmitter plays any role for PCa remains unanswered.

Blood flow is an essential physiologic parameter for cancer growth, and, hence, tumour blood flow (TBF) has been measured for characterization of tumours of various origins [3], amongst others in prostate-[15–17] lung-[18, 19], breast-[20–23], head and neck-[24], colorectal-[19, 25], and brain cancer [26].

[^82^Rb]Rb is an accessible clinical blood flow tracer for positron emission tomography (PET), which is used for myocardial blood flow measurement in many PET-Centres worldwide. As coincidental findings, increased [^82^Rb]Rb uptake was described in neuroendocrine tumours [27], breast-[28], lung-[29], and kidney cancer [30], amongst others. Recently, we validated [^82^Rb]Rb PET for TBF measurement in PCa [31, 32]. In a recent large clinical study, we consolidated the positive correlation between TBF and PCa aggressiveness and found [^82^Rb]Rb TBF superior to apparent diffusion coefficient (ADC) from multiparametric (mp) magnetic resonance imaging (MRI) in differentiating between clinically significant and insignificant PCa [33]. Inflammatory lesions displayed increased [^82^Rb]Rb TBF [33].

Both PSMA-uptake and TBF in PCa correlate with PCa aggressiveness. However, the two physiologic parameters have not previously been studied together. From our previous studies regarding TBF in clinical PCa patients, we have access to a sub-cohort, who underwent both [^68^Ga]Ga-PSMA-11- and [^82^Rb]Rb PET/computed tomography (CT). Hence, the aim of the present study was to evaluate whether the two physiologic parameters are associated and whether there is a synergistic benefit of combining them for separation of significant PCa from insignificant findings.

### Methods

#### Patient population

Across our previous studies [31–33], 43 patients underwent both pelvic [^82^Rb]Rb PET/CT and clinical [^68^Ga]Ga-PSMA-11 PET/CT and thereby met the inclusion criteria for the present study. All patients were recruited at the time of primary staging and hence none had previous therapy. The designs of the three studies from which the patients originated varied, and hence, the available ISUP GG derived from either radical prostatectomy, MRI-guided biopsies, or trans-rectal ultrasound-guided biopsies. Twenty-four patients with in total 33 lesions had MR-guided in-bore biopsy. Nineteen patients had trans-rectal ultrasound-guided biopsy, and 23 patients underwent subsequent radical prostatectomy. Histopathological inflammation for each lesion was registered.

The study was approved by the institutional review board (Central Denmark Region Committees on Health Research Ethics) and all subjects signed an informed consent form.

### Imaging

All [^82^Rb]Rb PET/CT scans but one were performed on GE Discovery MI Digital Ready PET/CT (GE Healthcare, Waukesha, Wisconsin, USA), and a single scan was performed on GE Discovery MI (5 ring) PET/CT. Details of the scan and reconstruction protocols have previously been described [31]. A bolus of [^82^Rb]RbCl (1110 MBq) was injected at scan start by the Cardiogen-82 generator infusion system (Bracco, Monroe Township, New Jersey, USA). The static image series of [^82^Rb]Rb PET (3 to7 min post-injection) were used for SUV analysis.

[^68^Ga]Ga-PSMA-11 PET/CT scans were performed one hour post-injection of 2.14 MBq [^68^Ga]Ga-PSMA-11 (^68^Ga-Glu-CO-Lys(Ahx)-HBED-CC) per kilogram body weight on a Siemens Biograph TruePoint PET/CT scanner (Siemens, Erlangen, Germany). 3D PET acquisitions with 3 min per bed position from vertex cranii to mid-femur were performed. Low-dose CT for attenuation correction was performed with all common corrections applied using the TrueX reconstruction algorithm (4 iterations and 21 subsets) and a 3-mm Gaussian post-filter (XYZ).

The mpMRI scans were performed according to clinical guidelines and the PIRADS v 2.1 minimum protocol [34] on 3 T platform Siemens Skyra (Siemens, Erlangen, Germany).

### Image analysis

[^68^Ga]Ga-PSMA-11 PET/CT scans and [^82^Rb]Rb PET/CT scans were co-registered using the CT scan as bridge. Co-registrations with the T2 weighted sequence of the mpMRI were also carried out in the 24 patients where mpMRI was available, again using the CT scan as bridge. Volume-of-interests (VOI) were defined in two different ways. First, the tumour VOIs were defined by the [^68^Ga]Ga-PSMA-11 activity. The threshold used for automatic VOI drawing on [^68^Ga]Ga-PSMA-11 PET varied between individual patients, as no universal threshold could be defined. In two patients with basal tumour location, the bladder activity was masked. An external tumour VOI defined by the mpMRI was applied in the 24 patients with mpMRI scan available. The tumour VOIs were transferred to the [^82^Rb]Rb PET/CT and/or [^68^Ga]Ga-PSMA-11 PET/CT scans for measurement of TBF and [^68^Ga]Ga-PSMA-11-uptake, respectively. The analyses were mainly performed on the data from [^68^Ga]Ga-PSMA-11-guided VOIs; forty-eight lesions could be automatically drawn from the [^68^Ga]Ga-PSMA-11 hotspot, whereas the last four lesions (benign and ISUP GG-1) displayed too low activity and was drawn manually, guided by MRI. The cohort with MRI-guided VOIs was analysed for using an external modality to test the models on less biased data.

Image co-registration and VOI analysis were performed with Hermes Viewer version 5.0 (Hermes Medical Solutions, Stockholm, Sweden).

### Statistical analysis

The ISUP GG used in analysis was a “best estimate”, using post-prostatectomy ISUP GG if available, otherwise MRI-guided biopsy ISUP GG if available and, if none of the previous were accessible, trans-rectal ultrasound-guided biopsy ISUP GG were used.

Q-Q-plots and histograms were used for testing data normality. Normally distributed data are reported as mean ± standard deviation and non-normally distributed data as median with range and log-transformation in parametric analysis. p values < 0.05 were considered statistically significant.

As ISUP GG is an ordinal scale, Spearman’s rank correlation (rho) was applied for analysis of correlations involving ISUP GG. For continuous variables, Pearson’s correlation (r) was applied. For correlation analysis, ISUP GG = 0 was used for benign lesions in some analyses. Receiver operating characteristic (ROC) analyses were used for calculating area under the curve (AUC), sensitivity and specificity. Negative predictive value (NPV) and positive predictive value (PPV) were calculated according to standard definitions. McNemar’s test for difference between paired nominal data was used to compare the diagnostic accuracies. The interaction between [^68^Ga]Ga-PSMA-11 SUVmax and [^82^Rb]Rb SUVmax for separation of ISUP GG was tested using ordinal and nominal logistic fit models.

Data were collected and managed using REDCap (Vanderbilt University Medical Center, Nashville, Tennessee, USA), hosted at Aarhus University [35]. Statistical analysis was performed in STATA version 16.1 (StataCorp LLC, College Station, Texas, USA) and JMP (SAS Institute Inc., Cary, North Carolina, United States).

## Results

Patient characteristics are found in Table 1. In total, 52 lesions from 43 patients were analysed. The “best estimate” ISUP GG consisted of 23 lesions with post-prostatectomy ISUP GG, 17 lesions with MRI-guided biopsy ISUP GG, and 12 lesions with trans-rectal ultrasound-guided biopsy ISUP GG. Histopathological inflammation was found in six.

**Table 1:** Patient characteristics. Numbers represent median [range]

	All patients n = 43
Age (Years)	69 [51; 79]
PSA (ng/mL)	13.5 [4.7; 168.6]

**Fig. 1:**
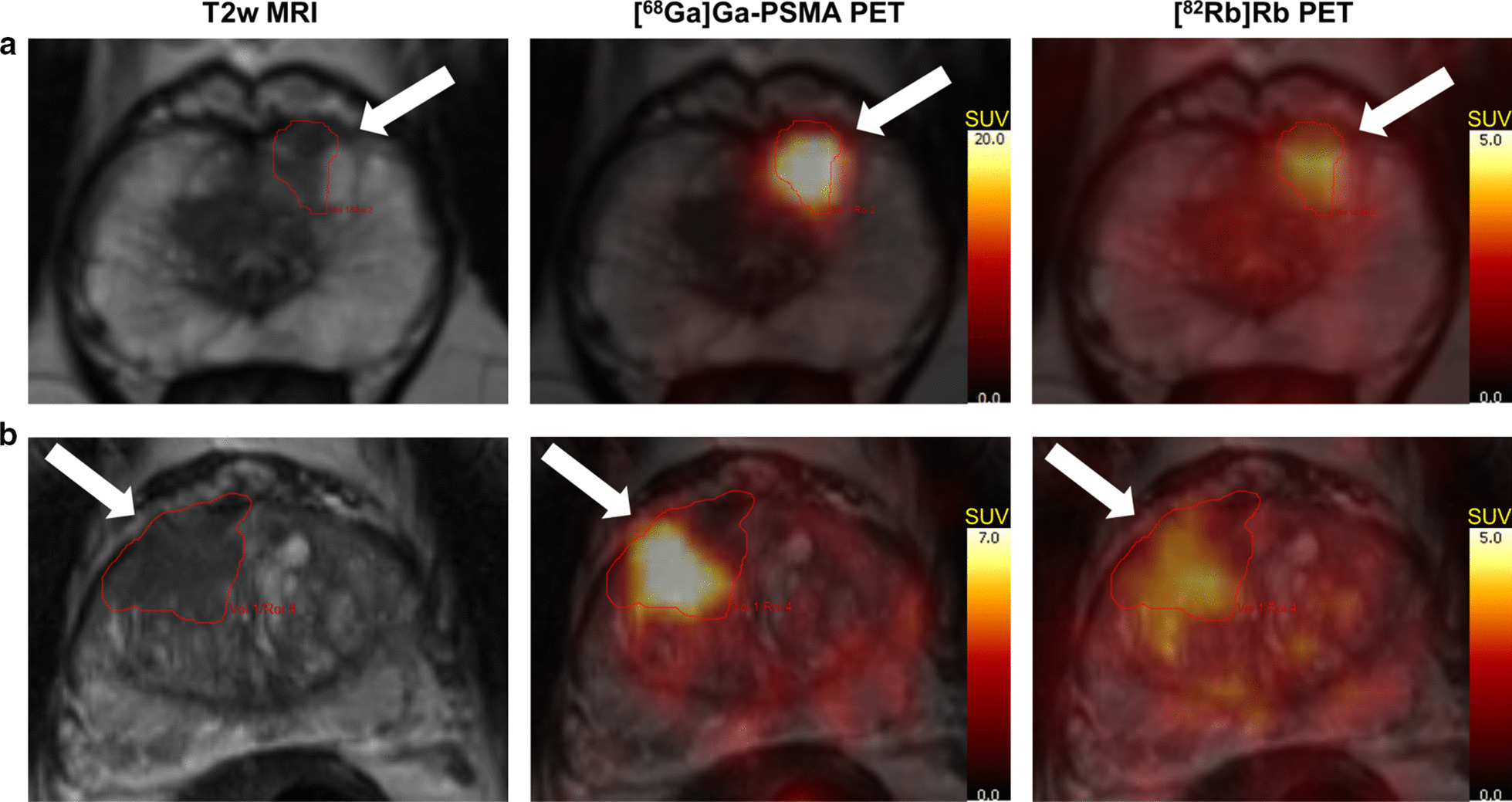
Demonstration of a MRI-guided tumour VOI with similar high uptake patterns of both [^68^Ga]Ga-PSMA-11 and [^82^Rb]Rb (**a**) and an example of inhomogeneous tracer distribution within the MRI-guided tumour VOI (**b**)

The distribution of patients in different ISUP GG’s is displayed in Table 2. The heterogeneity of the cohort is caused by consecutive recruitment prior to primary histological staging for most patients. The tumour delineation was congruent on MRI, [^68^Ga]Ga-PSMA-11 and [^82^Rb]Rb PET in many cases as shown in Fig. 1a. However, as shown in Fig. 1b, [^68^Ga]Ga-PSMA-11-uptake and TBF could be inhomogeneous within the MRI-guided tumour VOI. In other cases, the [^82^Rb]Rb- and/or the [^68^Ga]Ga-PSMA-11-activity extended beyond the border of the MRI VOI.

**Table 2:** [^68^Ga]Ga-PSMA-11 and [^82^Rb]Rb uptake per ISUP GG including correlations. * Benign lesions are included as ISUP GG = 0

Tracer	Measure	Benign (n = 2)	ISUP Grade Group					Correlations	
			1 (n = 4)	2 (n = 21)	3 (n = 10)	4 (n = 6)	5 (n = 9)	ISUP GG (n = 50)	ISUP GG* (n = 52)
[^68^Ga]Ga-PSMA-11	SUVmax	3.04 [2.5; 3.6]	5.54 [3.7; 26.4]	8.03 [5.1; 36.5]	22.39 [13.8; 80.1]	27.76 [9.3; 41.7]	32.23 [5.5; 61.8]	rho = 0.54 p < 0.001	rho = 0.59 p < 0.001
	SUVmean	1.55 [1.3; 1.8]	3.47 [1.7; 10.6]	5.22 [3.5; 12.5]	8.51 [7.0; 22.1]	10.06 [6.4; 14.5]	8.60 [3.1; 18.7]	rho = 0.50 p < 0.001	rho = 0.55 p < 0.001
	SUVpeak	1.94 [1.7; 2.2]	2.84 [1.9; 18.3]	4.31 [2.7; 18.9]	10.19 [6.6; 55.4]	14.79 [5.3; 28.7]	12.47 [3.0; 43.7]	rho = 0.51 p < 0.001	rho = 0.56 p < 0.001
[^82^Rb]Rb	SUVmax	1.99 ± 0.06	3.29 ± 1.11	3.67 ± 0.78	4.86 ± 1.25	4.90 ± 0.99	4.91 ± 1.59	rho = 0.50 p < 0.001	rho = 0.56 p < 0.001
	SUVmean	1.47 ± 0.08	2.21 ± 0.81	2.60 ± 0.57	2.87 ± 0.50	3.06 ± 0.57	2.91 ± 0.59	rho = 0.35 p = 0.01	rho = 0.42 p = 0.002
	SUVpeak	1.63 ± 0.08	2.78 ± 0.98	2.90 ± 0.56	3.73 ± 0.92	3.94 ± 0.97	3.80 ± 0.95	rho = 0.49 p < 0.001	rho = 0.55 p < 0.001

Median values for [^68^Ga]Ga-PSMA-11 SUV-measures and mean values of [^82^Rb]Rb SUV-measures alongside correlations with ISUP GG are provided in Table 2.

lesions.

**Fig. 2:**
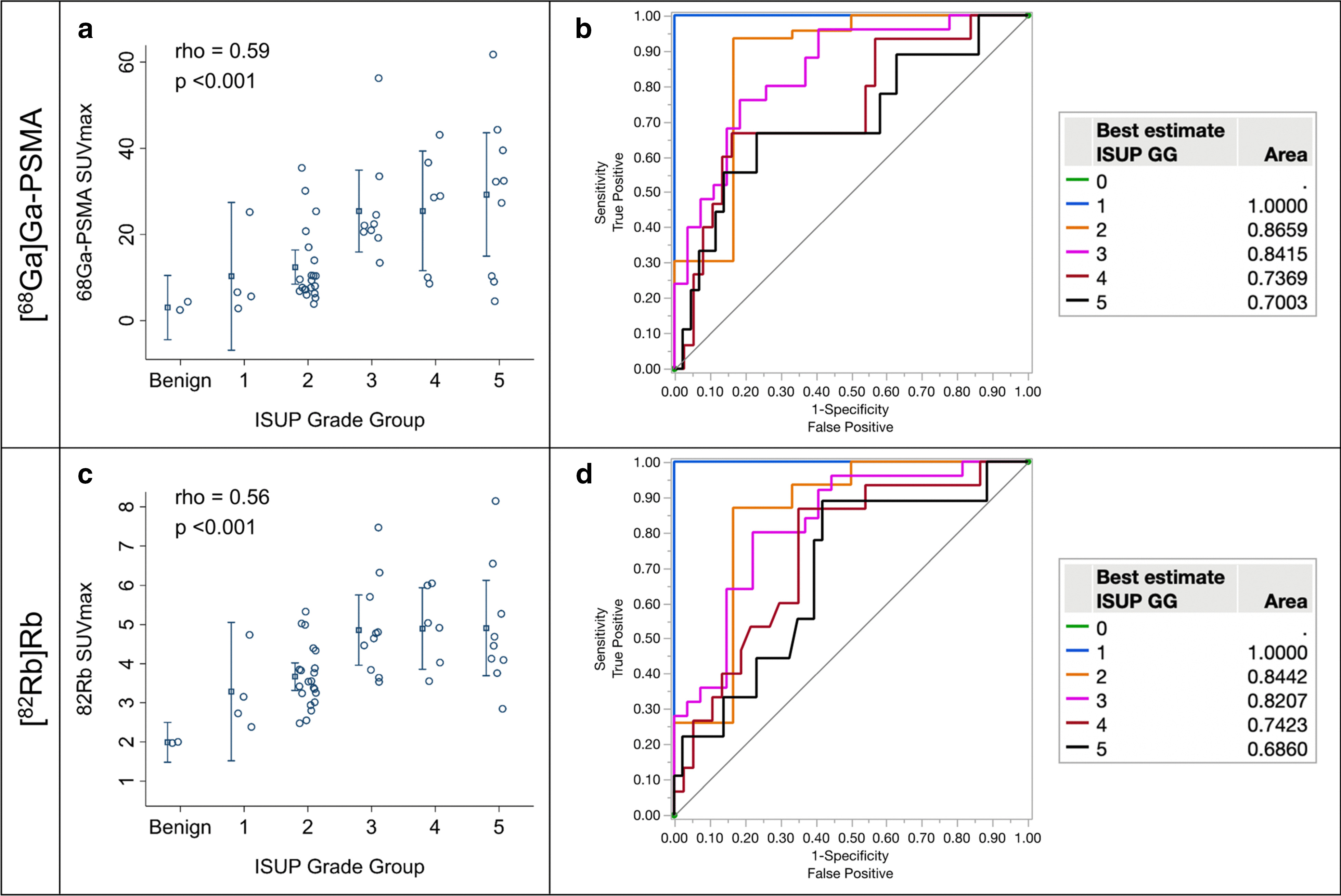
Plots of [^68^Ga]Ga-PSMA-11 SUVmax against ISUP GG (**a**) and [^82^Rb]Rb SUVmax against ISUP GG (**c**). An outlier (ISUP GG-3, SUVmax = 80) was excluded from the plot in (**a**) to minimize the extension of the y-axis. Bars are mean (blue square) with confidence interval (whiskers). ROC curves for [^68^Ga]Ga-PSMA-11 SUVmax and [^82^Rb]Rb SUVmax for the ability to separate at each ISUP GG (**b**, **d**)

[^68^Ga]Ga-PSMA-11 SUVmax and [^82^Rb]Rb SUVmax are plotted against ISUP GG in Fig. 2a and 2c, respectively. ROC curves for separation based on "best estimate” ISUP GG are found in Fig. 2b and 2d for [^68^Ga]Ga-PSMA-11 SUVmax and [^82^Rb]Rb SUVmax, respectively. The results of the ROC analyses are found in Tables 3 and 4. The “model” used in these tables is extremely simple, if either the [^68^Ga]Ga-PSMA-11 SUVmax or [^82^Rb]Rb SUVmax is above their cut-off this results in a positive test. In contrast, if both are below their cut-off this results in a negative test. ISUP GG-0 and GG-1 consist of only six lesions in total, which makes the results in Table 3 uncertain.

**Table 3:** ROC analysis data for separation of ISUP GG > 1 from GG-1 and benign lesions for [^68^Ga]Ga-PSMA-11 SUVmax and [^82^Rb]Rb SUVmax

Measure	Cut-off	AUC	Sensitivity	Specificity	Correctly classified	False negative	False positive	Negative predictive value	Positive predictive value
[^68^Ga]Ga-PSMA-11 SUVmax	5.75	0.87	93%	83%	48	3	1	0.63	0.98
[^82^Rb]Rb SUVmax	3.21	0.84	87%	83%	45	6	1	0.45	0.98
Combined model	PSMA = 5.75 [^82^Rb]Rb = 3.21	0.89	98%	83%	50	1	1	0.83	0.98

**Table 4:** ROC analysis data for separation of ISUP GG > 2 from GG-1–2 and benign lesions for [^68^Ga]Ga-PSMA-11 SUVmax, ^82^Rb SUVmax, and the combined model

Measure	Cut-off	AUC	Sensitivity	Specificity	Correctly classified	False negative	False positive	Negative predictive value	Positive predictive value
[^68^Ga]Ga-PSMA-11 SUVmax	18.78	0.84	76%	81%	41	6	5	0.79	0.79
[^82^Rb]Rb SUVmax	3.90	0.82	80%	78%	41	5	6	0.81	0.77
Combined model	PSMA = 18.78 [^82^Rb]Rb = 3.90	0.85	96%	74%	44	1	7	0.95	0.77

**Fig. 3:**
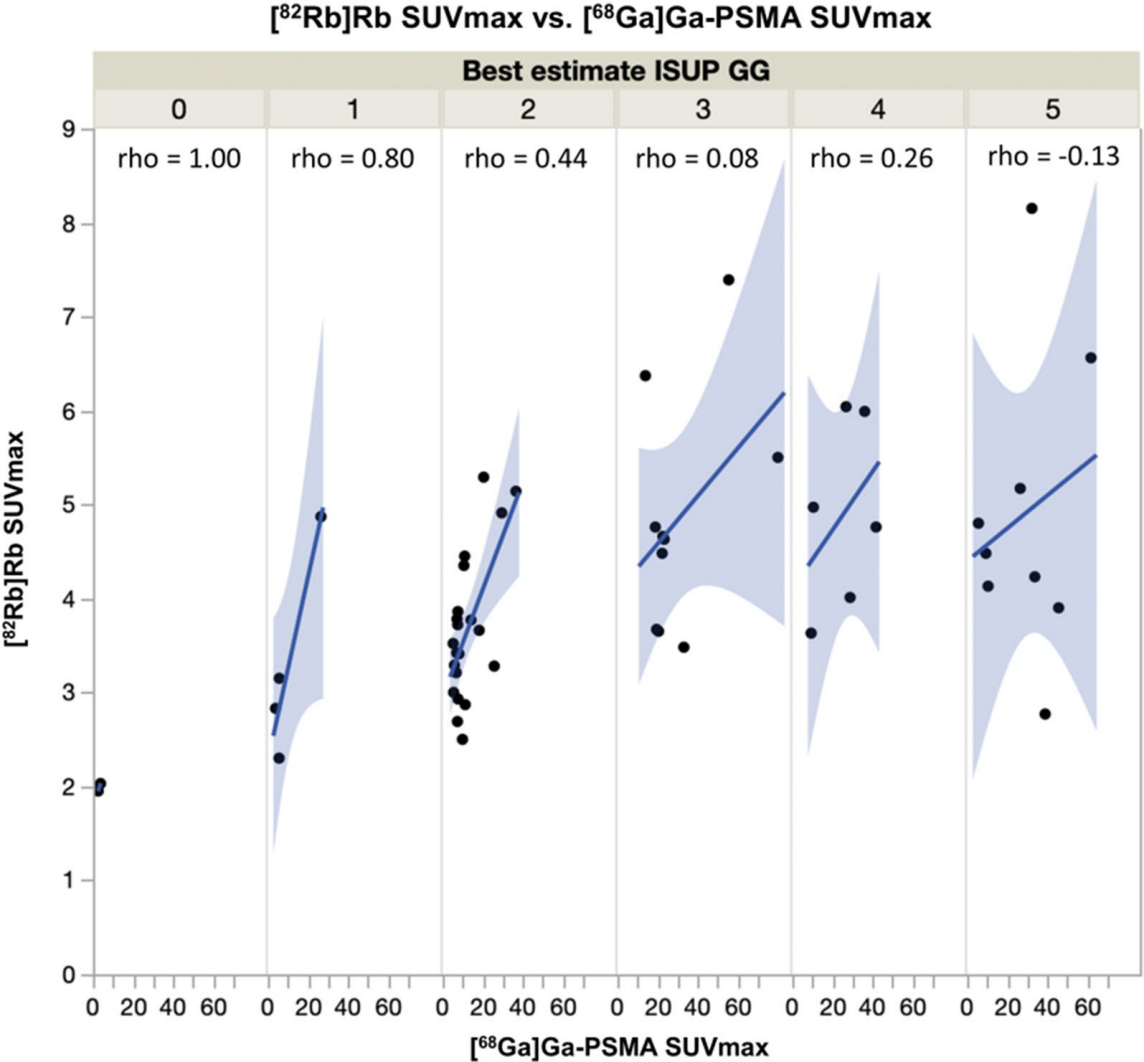
Plot of tumour [^68^Ga]Ga-PSMA-11 SUVmax against [^82^Rb]Rb SUVmax for each ISUP GG

[^68^Ga]Ga-PSMA-11 SUVmax (log-transformed) and [^82^Rb]Rb SUVmax were correlated (Pearson’s r = 0.65, p < 0.001). The correlations for SUVmean and SUVpeak were equivalent. As shown in Fig. 3, the correlation was especially evident in the lower risk groups (ISUP GG-1–2), whereas it faded with increasing ISUP GG. The pattern for log-transformed data was equivalent.

**Fig. 4:**
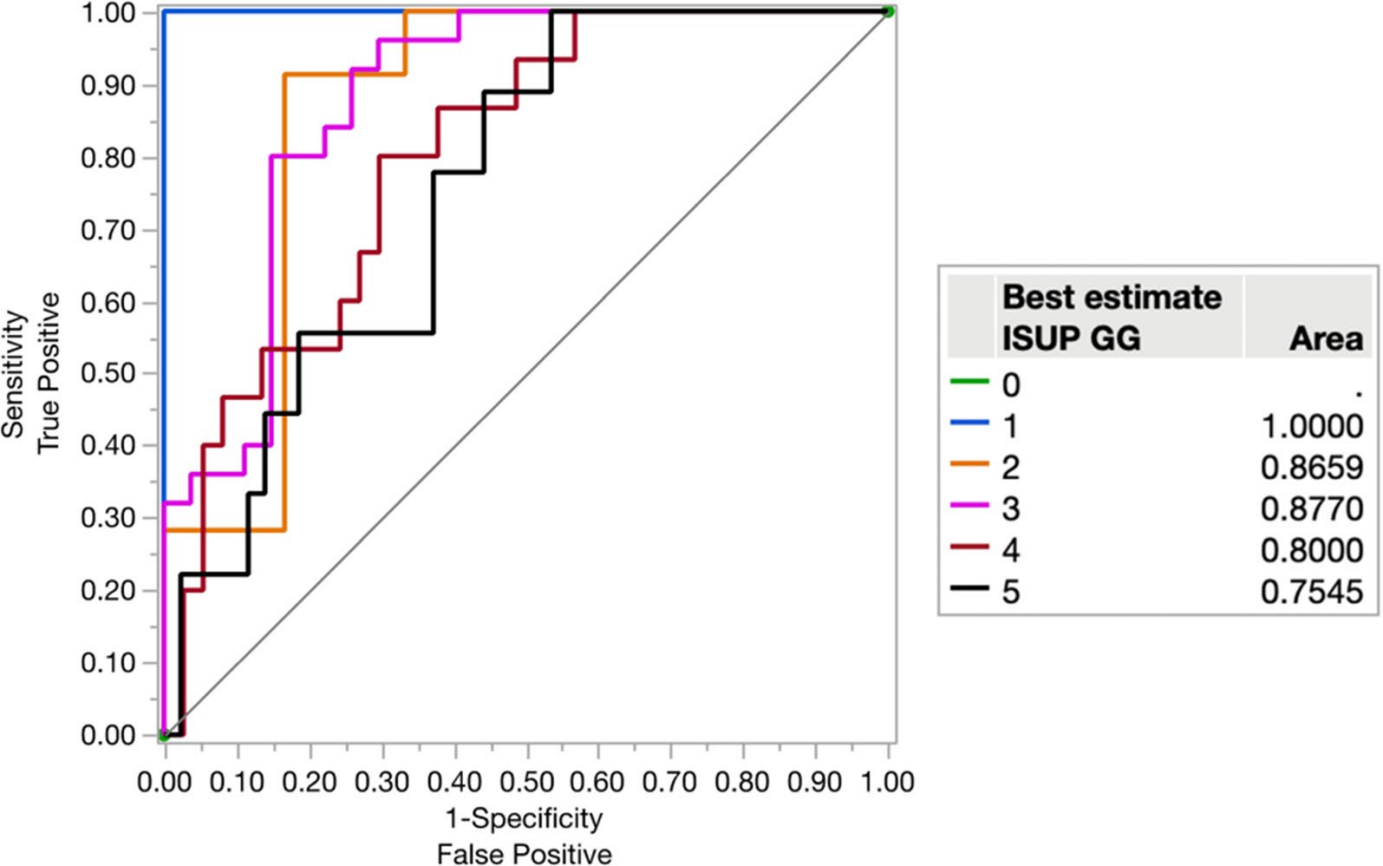
ROC curves for the combined model of [^68^Ga]Ga-PSMA-11 SUVmax and [^82^Rb]Rb SUVmax to separate at each ISUP GG

We tested a combined model of [^68^Ga]Ga-PSMA-11 SUVmax and [^82^Rb]Rb SUVmax for separation of ISUP GG (Fig. 4). Both parameters were highly significant (p < 0.001), meaning that both parameters contributed to the model, and as a result, the NPV improved significantly for separation of ISUP GG > 2 from ISUP GG1-2 and benign lesions, without compromising the PPV (Table 4). The interaction parameter between [^68^Ga]Ga-PSMA-11 SUVmax and [^82^Rb]Rb SUVmax for separation of ISUP GG was highly significant as well (p < 0.001). McNemar’s tests for difference between the combined analysis and [^68^Ga]Ga-PSMA-11 SUVmax alone (p = 0.008) and [^82^Rb]Rb SUVmax alone (p = 0.025) to separate ISUP GG > 2 from ISUP GG ≤ 2 and benign lesions were significant.

The model was subsequently tested on the sub-cohort with MRI-guided VOIs, considering the mpMRI as an external modality, to minimize the bias introduced by defining VOIs based on [^68^Ga]Ga-PSMA-11 hotspots. Furthermore, analysis with weight for the number of lesions per patient and the removal of multiple lesions per patient were performed. Analysis with registration of the inflammatory lesions was performed. Neither of these control analyses affected the results.

**Fig. 5:**
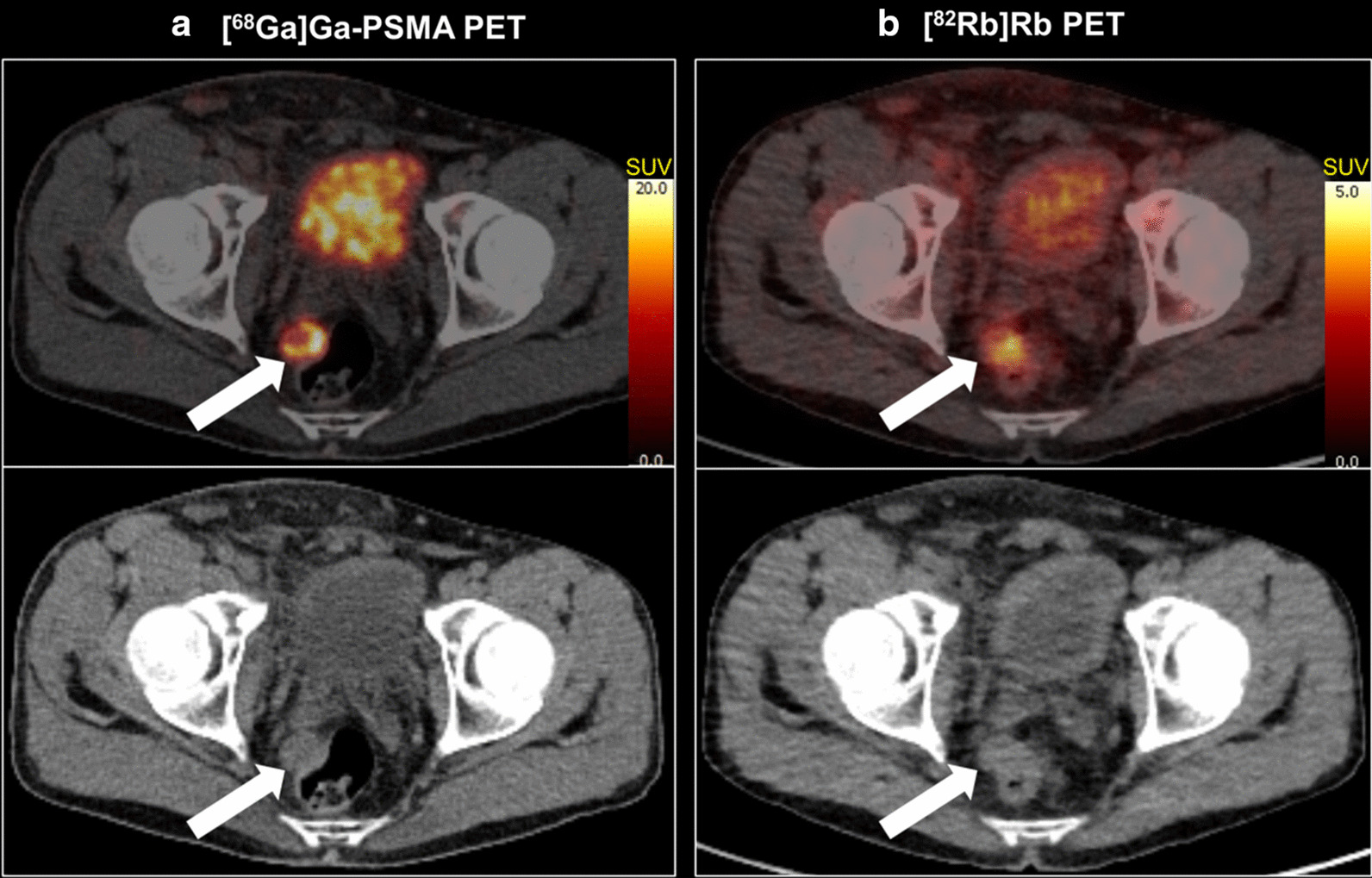
Large pararectal lymph node metastasis with both highly increased [^68^Ga]Ga-PSMA-11 uptake (**a**) and [^82^Rb]Rb uptake (**b**) on transaxial fused PET/CT images

**Fig. 6:**
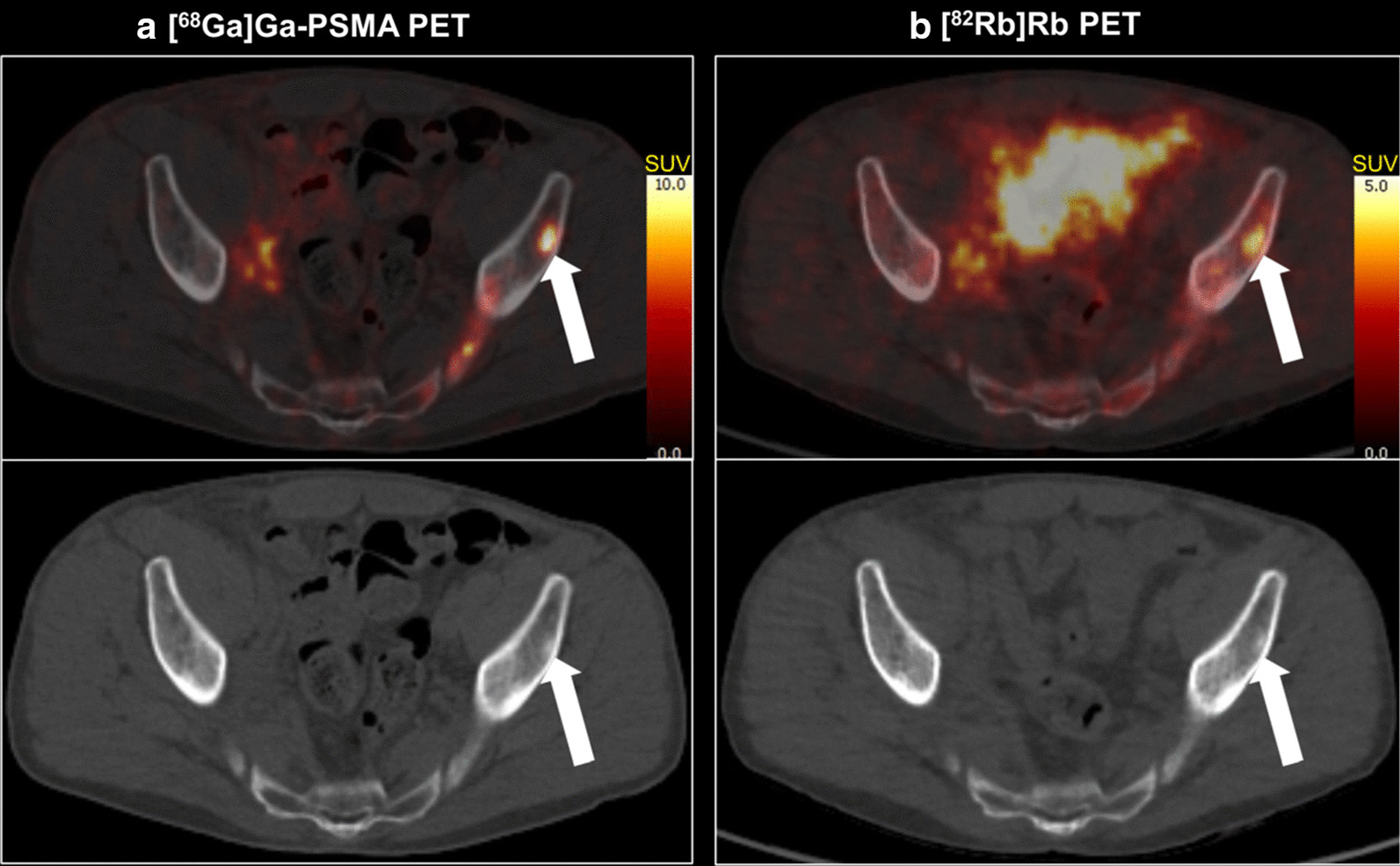
Bone metastasis in the left iliac bone with avid [^68^Ga]Ga-PSMA-11 uptake (**a**) and highly increased blood flow in the metastatic area (**b**). The bone window of corresponding low-dose CT reveals slightly enhanced bone density (**a**, **b**)

Soft tissue metastases as well as bone metastases with increased [^68^Ga]Ga-PSMA-11 uptake and increased blood flow were detected (shown in Figs. 5, 6).

## Discussion

The main results of the present study are that tumour [^68^Ga]Ga-PSMA-11 uptake and [^82^Rb]Rb TBF are associated and that both correlate with PCa aggressiveness. The present study suggests that a combined analysis of [^68^Ga]Ga-PSMA-11 uptake and TBF provides a synergetic effect that reduces the number of false negatives and gives a high accuracy for separation of clinically significant PCa from insignificant findings.

### [^68^Ga]Ga-PSMA-11-uptake for prediction of PCa aggressiveness

The correlation between [^68^Ga]Ga-PSMA-11 SUVmax and ISUP GG varies greatly across the published studies. The present correlation is in line with the study by Chen et al. (rho = 0.55, p < 0.01, n = 51, radical prostatectomy Gleason Score) [8] and notably higher than in previous studies by our group (rho = 0.21, p < 0.001, n = 690, for biopsy ISUP GG) and (rho = 0.38, p < 0.001, n = 247, radical prostatectomy ISUP GG) [10] and by Cytawa et al. (rho = 0.35, p = 0.001, n = 82, biopsy Gleason Score) [9]. Another study by Demirci et al. found a good correlation (r = 0.66, p < 0.001) between SUVmax and ISUP GG in 141 patients [6]; however, they used Pearson’s correlation, which should not be applied for ordinal variables such as ISUP GG. A direct comparison between the above-mentioned studies is challenging, as some studies used Gleason Score while others used ISUP GG, Klingenberg et al. [10] included high-risk patients only, where the correlation seemed to disappear in the present study, and finally Cytawa et al. [9] used the [^68^Ga]Ga-PSMA-I&T ligand.

The AUC, sensitivity and specificity for [^68^Ga]Ga-PSMA-11 SUVmax in the present study (Table 4) were in line with the results from Demirci et al. [6], and again, substantially higher than the results from Klingenberg et al. [10]. A direct comparison is, again, not fair, as Klingenberg et al. examined high-risk patients exclusively. Meanwhile, the large overlap between the ISUP GGs, where even very aggressive ISUP GG-4–5 tumours can display very low [^68^Ga]Ga-PSMA-11 uptake, are also seen in the present study (Fig. 2a).

Tumour delineation is not always identical between the three modalities used in the present study, as the MRI VOI is occasionally expanded, especially by the [^68^Ga]Ga-PSMA-11 activity. As proposed by Zamboglou et al. [36], this could mean that the tumour delineation is more precise if not based on a sole modality. Hence, tumour delineation for radiation planning [36] and biopsy guidance [37] could be potential indications for [^68^Ga]Ga-PSMA-11 PET/MRI.

### Tumour blood flow for prediction of PCa aggressiveness

The coherency between quantitative TBF and PCa aggressiveness is being established, and the present results are in line with our previous publications [31, 33]. However, the potential clinical role of quantitative TBF is undetermined in PCa management. The results of TBF measurement with [^82^Rb]Rb complements other studies using quantitative pharmacokinetic analysis of the dynamic contrast-enhanced series of MRI, which found an improvement in the PPV of mpMRI for primary local staging of PCa [38–41]. Meanwhile, the studies using the simpler qualitative analysis of the dynamic contrast-enhanced series generally report more limited additional value of the Gadolinium contrast examination [42]. Hence, there could be an unexploited potential in the quantitative pharmacokinetic analysis of prostate dynamic contrast-enhanced MRI at selecting which patients to biopsy, as absolute quantification of perfusion with DCE MRI might provide comparable information to perfusion PET. In PET studies on other cancers though, low blood flow—high metabolism mismatch, which is essentially a sign of hypoxia, was found to be the best predictor of tumour grade, treatment response, and/or survival [43–46].

Studies showed that the decrease in TBF as response to neoadjuvant chemotherapy in locally advanced breast cancer was a strong predictor of disease-free survival and overall survival [21, 23] and that TBF change in metastases from different primary tumours after sunitinib malate treatment was predictive of clinical benefit [19]. Another study showed that the change in TBF provides information on anti-angiogenic treatment response beyond prostate-specific antigen (PSA) in androgen independent PCa [15]. Hence, another potential gain could be to measure changes in TBF over time, for example during therapy or during active surveillance.

### Potential synergy between PSMA uptake and TBF for prediction of significant PCa

Even though both PSMA uptake and TBF can classify most lesions correctly into significant or non-significant PCa, the accuracies of the methods individually are probably too low for clinical decision-making. The main limitation for both methods is the number of false negatives, which means that clinically significant PCa are missed. It is well known that cancers become increasingly heterogeneous as they dedifferentiate and becomes more aggressive. This heterogeneity regards PSMA expression [47]] and apparently, TBF too, as both parameters can occasionally be low in ISUP GG-4 and GG-5 patients (Fig. 2a, c). Due to the complex tumour biology, a single aspect of physiology seems too sparse for full separation of significant from insignificant PCa. The highly significant interaction parameter found in the present study shows a crossed interaction between PSMA uptake and TBF. This means that PSMA uptake and TBF are complementary in some of the high-risk patients, where the correlation between the two parameters is lost (Fig. 3). By utilizing this complementary tumour biology information with a combined analysis using both PSMA uptake and TBF, especially the NPV for separation of clinically significant PCa from insignificant findings improves, without compromising the PPV. This results in a significant reduction in the false negatives (missed significant PCa) from six to one in this cohort (Table 4). The model, including the interaction parameter, was significant regardless of VOI drawing methods and compensation for multiple lesions per patient. The model is preliminary though and needs further external validation in larger studies, but the basic principles should be generalizable. Such multiparametric PET approach may be the key to non-invasive risk evaluation. These results illustrate that two aspects of tumour biology characterizes the tumour more precisely than one. A similar synergy with other physiologic parameters, hypoxia for example, may very well show the same tendency. An advantage is that PSMA PET and TBF are very much within clinical reach, as PSMA PET is already a dominating imaging modality in PCa imaging, and TBF can be estimated with various modalities. Other studies found correlations between PCa aggressiveness and K_1_ influx of other tracers including [^15^O]-H_2_O [17], [^18^F]-flourocholine [48, 49], [^11^C]-donepezil [50], and [^11^C]-acetate [51]. The authors believe that the most promising applications of this combined analysis is characterization of tumour metabolism for primary risk evaluation and monitoring more than for detection of biochemical recurrence.

We found it important that the two tracers performed with such similarity in the analyses. The correlation between tumour [^68^Ga]Ga-PSMA-11- and [^82^Rb]Rb uptake suggests that the tumour PSMA uptake may be flow-limited (Fig. 3). We found this sufficiently interesting to design a future study with dynamic PSMA PET and ^15^O-H_2_O PET, which is gold standard of perfusion, to characterize the composition of the PSMA signal. If the PSMA uptake rate is an appropriate reflection of TBF in PCa, an approach with early dynamic plus late static PSMA PET scanning may examine both PSMA uptake and TBF and provide valuable additional information of biological potential of the tumour in selected patient categories. This could be a further step towards PSMA PET as a one-stop shop in PCa imaging to assess both whole body tumour burden and biological potential of the primary tumour.

## Conclusions

PSMA uptake and TBF both correlate with PCa aggressiveness, but the number of false negatives in separating significant from insignificant PCa makes the methods insufficient for clinical use as a sole risk stratification parameter. The present study demonstrated an association between PSMA uptake and TBF, but that they also provided complementary insights into tumour biology. Thus, the present study suggests that a combined assessment of PSMA uptake and TBF could significantly reduce the number of false negatives and, hence, allow non-invasive separation of significant from insignificant PCa.

## Data Availability

The dataset supporting the conclusions of this article is included within the article.

## Abbreviations

ADC: Apparent diffusion coefficient
AUC: Area under the curve
CT: Computed tomography
GG: Grade group
ISUP: International Society of Urological Pathology
mp: Multiparametric
MRI: Magnetic resonance imaging
PCa: Prostate cancer
PET: Positron emission tomography
PIRADS: Prostate imaging-reporting and data system
PSA: Prostate-specific antigen
PSMA: Prostate-specific membrane antigen
Rb: Rubidium
ROC: Receiver operating characteristics
SUV: Standardized uptake value
TBF: Tumour blood flow
VOI: Volume of interest

## References

[1] Attard G, Parker C, Eeles RA, Schroder F, Tomlins SA, Tannock I (2016). Prostate cancer. Lancet (London, England).

[2] Hanahan D, Weinberg RA (2011). Hallmarks of cancer: the next generation. Cell.

[3] Johnson GB, Harms HJ, Johnson DR, Jacobson MS (2020). PET Imaging of Tumor Perfusion: A Potential Cancer Biomarker?. Semin Nucl Med.

[4] Hofman MS, Lawrentschuk N, Francis RJ, Tang C, Vela I, Thomas P (2020). Prostate-specific membrane antigen PET-CT in patients with high-risk prostate cancer before curative-intent surgery or radiotherapy (proPSMA): a prospective, randomised, multicentre study. The Lancet.

[5] Ferdinandus J, Fendler WP, Hadaschik B, Herrmann K (2020). Prostate-specific membrane antigen targeted PET imaging for prostate cancer recurrence. Curr Opin Urol.

[6] Demirci E, Kabasakal L, Sahin OE, Akgun E, Gultekin MH, Doganca T (2019). Can SUVmax values ofGa-68-PSMA PET/CT scan predict the clinically significant prostate cancer?. Nucl Med Commun.

[7] Uprimny C, Kroiss AS, Decristoforo C, Fritz J, von Guggenberg E, Kendler D (2017). (68)Ga-PSMA-11 PET/CT in primary staging of prostate cancer: PSA and Gleason score predict the intensity of tracer accumulation in the primary tumour. Eur J Nucl Med Mol Imaging.

[8] Chen M, Qiu X, Zhang Q, Zhang C, Zhou Y, Zhao X, et al. PSMA uptake on [68Ga]-PSMA-11-PET/CT positively corrects with prostate cancer aggressiveness. The quarterly journal of nuclear medicine and molecular imaging : official publication of the Italian Association of Nuclear Medicine (AIMN) [and] the International Association of Radiopharmacology (IAR), [and] Section of the So. 2019.

[9] Cytawa W, Seitz AK, Kircher S, Fukushima K, Tran-Gia J, Schirbel A (2020). (68)Ga-PSMA I&T PET/CT for primary staging of prostate cancer. Eur J Nucl Med Mol Imaging.

[10] Klingenberg S, Jochumsen MR, Ulhøi BP, Fredsøe J, Sørensen KD, Borre M, et al. (68)Ga-PSMA PET/CT for primary NM staging of high-risk prostate cancer. Journal of nuclear medicine : official publication, Society of Nuclear Medicine. 2020. 10.2967/jnumed.120.245605 32444374

[11] Zhou J, Neale JH, Pomper MG, Kozikowski AP (2005). NAAG peptidase inhibitors and their potential for diagnosis and therapy. Nat Rev Drug Discovery.

[12] Yao V, Bacich DJ. Prostate specific membrane antigen (PSMA) expression gives prostate cancer cells a growth advantage in a physiologically relevant folate environment in vitro. 2006;66(8):867–75. 10.1002/pros.20361 16496414

[13] Yao V, Berkman CE, Choi JK, O'Keefe DS, Bacich DJ. Expression of prostate-specific membrane antigen (PSMA), increases cell folate uptake and proliferation and suggests a novel role for PSMA in the uptake of the non-polyglutamated folate, folic acid. 2009:n/a-n/a. 10.1002/pros.21065 19830782

[14] Ghosh A, Wang X, Klein E, Heston WD (2005). Novel role of prostate-specific membrane antigen in suppressing prostate cancer invasiveness. Can Res.

[15] Kurdziel KA, Figg WD, Carrasquillo JA, Huebsch S, Whatley M, Sellers D (2003). Using positron emission tomography 2-deoxy-2-[18F]fluoro-D-glucose, 11CO, and 15O-water for monitoring androgen independent prostate cancer. MIB.

[16] Inaba T (1992). Quantitative measurements of prostatic blood flow and blood volume by positron emission tomography. J Urol.

[17] Tolbod LP, Nielsen MM, Pedersen BG, Hoyer S, Harms HJ, Borre M (2018). Non-invasive quantification of tumor blood flow in prostate cancer using (15)O-H2O PET/CT. Am J Nucl Med Mol Imaging.

[18] de Langen AJ, van den Boogaart V, Lubberink M, Backes WH, Marcus JT, van Tinteren H (2011). Monitoring response to antiangiogenic therapy in non-small cell lung cancer using imaging markers derived from PET and dynamic contrast-enhanced MRI. J Nucl Med.

[19] Scott AM, Mitchell PL, O'Keefe G, Saunder T, Hicks RJ, Poon A (2012). Pharmacodynamic analysis of tumour perfusion assessed by 15O-water-PET imaging during treatment with sunitinib malate in patients with advanced malignancies. EJNMMI Res.

[20] Krak N, van der Hoeven J, Hoekstra O, Twisk J, van der Wall E, Lammertsma A (2008). Blood flow and glucose metabolism in stage IV breast cancer: heterogeneity of response during chemotherapy. MIB.

[21] Mankoff DA, Dunnwald LK, Gralow JR, Ellis GK, Schubert EK, Tseng J (2003). Changes in blood flow and metabolism in locally advanced breast cancer treated with neoadjuvant chemotherapy. J Nucl Med.

[22] Specht JM, Kurland BF, Montgomery SK, Dunnwald LK, Doot RK, Gralow JR (2010). Tumor metabolism and blood flow as assessed by positron emission tomography varies by tumor subtype in locally advanced breast cancer. Clin Cancer Res.

[23] Dunnwald LK, Gralow JR, Ellis GK, Livingston RB, Linden HM, Specht JM (2008). Tumor metabolism and blood flow changes by positron emission tomography: relation to survival in patients treated with neoadjuvant chemotherapy for locally advanced breast cancer. J Clin Oncol.

[24] Lehtio K, Oikonen V, Gronroos T, Eskola O, Kalliokoski K, Bergman J (2001). Imaging of blood flow and hypoxia in head and neck cancer: initial evaluation with [(15)O]H(2)O and [(18)F]fluoroerythronitroimidazole PET. J Nucl Med.

[25] Lubberink M, Golla SS, Jonasson M, Rubin K, Glimelius B, Sorensen J (2015). (15)O-Water PET Study of the Effect of Imatinib, a Selective Platelet-Derived Growth Factor Receptor Inhibitor, Versus Anakinra, an IL-1R Antagonist, on Water-Perfusable Tissue Fraction in Colorectal Cancer Metastases. J Nucl Med.

[26] Bruehlmeier M, Roelcke U, Schubiger PA, Ametamey SM (2004). Assessment of hypoxia and perfusion in human brain tumors using PET with 18F-fluoromisonidazole and 15O–H2O. J Nucl Med.

[27] Hasbak P, Enevoldsen LH, Fosbøl MØ, Skovgaard D, Knigge UP, Kjær A (2016). Rubidium-82 uptake in metastases from neuroendocrine tumors: No flow response to adenosine. J Nucl Cardiol.

[28] Lu Y (2015). FDG and (82)Rb PET/MRI features of brain metastasis of breast cancer. Clin Nucl Med.

[29] Mirpour S, Khandani AH (2011). Extracardiac abnormalities on rubidium-82 cardiac positron emission tomography/computed tomography. Nucl Med Commun.

[30] Murthy VL, Brown RK, Corbett JR (2014). Metastatic renal cell carcinoma avid for 82Rb but not 18F-FDG. Clin Nucl Med.

[31] Jochumsen MR, Tolbod LP, Pedersen BG, Nielsen MM, Hoyer S, Frokiaer J (2019). Quantitative tumor perfusion imaging with (82)Rb PET/CT in prostate cancer: analytic and clinical validation. J Nucl Med.

[32] Jochumsen MR, Bouchelouche K, Nielsen KB, Frokiaer J, Borre M, Sorensen J (2019). Repeatability of tumor blood flow quantification with (82)Rubidium PET/CT in prostate cancer - a test-retest study. EJNMMI research.

[33] Jochumsen MR, Sörensen J, Pedersen BG, Nyengaard JR, Krag SRP, Frøkiær J, et al. Tumour blood flow for prediction of human prostate cancer aggressiveness: a study with Rubidium-82 PET, MRI and Na(+)/K(+)-ATPase-density. Eur J Nucl Med Mol Imaging. 2020. 10.1007/s00259-020-04998-2 PMC7835182 32808078

[34] Turkbey B, Rosenkrantz AB, Haider MA, Padhani AR, Villeirs G, Macura KJ, et al. Prostate Imaging Reporting and Data System Version 2.1: 2019 Update of Prostate Imaging Reporting and Data System Version 2. European urology. 2019;76(3):340–51. 10.1016/j.eururo.2019.02.033 30898406

[35] Harris PA, Taylor R, Thielke R, Payne J, Gonzalez N, Conde JG (2009). Research electronic data capture (REDCap)–a metadata-driven methodology and workflow process for providing translational research informatics support. J Biomed Inform.

[36] Zamboglou C, Drendel V, Jilg CA, Rischke HC, Beck TI, Schultze-Seemann W (2017). Comparison of (68)Ga-HBED-CC PSMA-PET/CT and multiparametric MRI for gross tumour volume detection in patients with primary prostate cancer based on slice by slice comparison with histopathology. Theranostics.

[37] Donato P, Morton A, Yaxley J, Ranasinghe S, Teloken PE, Kyle S (2020). (68)Ga-PSMA PET/CT better characterises localised prostate cancer after MRI and transperineal prostate biopsy: Is (68)Ga-PSMA PET/CT guided biopsy the future?. Eur J Nucl Med Mol Imaging.

[38] Cristel G, Esposito A, Damascelli A, Briganti A, Ambrosi A, Brembilla G (2019). Can DCE-MRI reduce the number of PI-RADS vol 2 false positive findings? Role of quantitative pharmacokinetic parameters in prostate lesions characterization. Eur J Radiol.

[39] Vos EK, Litjens GJ, Kobus T, Hambrock T, Hulsbergen-van de Kaa CA, Barentsz JO, et al. Assessment of prostate cancer aggressiveness using dynamic contrast-enhanced magnetic resonance imaging at 3 T. European urology. 2013;64(3):448–55. 10.1016/j.eururo.2013.05.045 23751135

[40] Hotker AM, Mazaheri Y, Aras O, Zheng J, Moskowitz CS, Gondo T (2016). Assessment of prostate cancer aggressiveness by use of the combination of quantitative DWI and dynamic contrast-enhanced MRI. AJR Am J Roentgenol.

[41] Chen YJ, Chu WC, Pu YS, Chueh SC, Shun CT, Tseng WY (2012). Washout gradient in dynamic contrast-enhanced MRI is associated with tumor aggressiveness of prostate cancer. J Magn Reson Imaging.

[42] Stabile A, Giganti F, Kasivisvanathan V, Giannarini G, Moore CM, Padhani AR, et al. Factors Influencing Variability in the Performance of Multiparametric Magnetic Resonance Imaging in Detecting Clinically Significant Prostate Cancer: A Systematic Literature Review. European Urology Oncology. 2020. 10.1016/j.euo.2020.02.005 PMC8942295 32192942

[43] Apostolova I, Hofheinz F, Buchert R, Steffen IG, Michel R, Rosner C, et al. Combined measurement of tumor perfusion and glucose metabolism for improved tumor characterization in advanced cervical carcinoma. A PET/CT pilot study using [15O]water and [18F]fluorodeoxyglucose. Strahlentherapie und Onkologie : Organ der Deutschen Rontgengesellschaft [et al]. 2014;190(6):575–81. 10.1007/s00066-014-0611-7 24535649

[44] Komar G, Kauhanen S, Liukko K, Seppänen M, Kajander S, Ovaska J (2009). Decreased blood flow with increased metabolic activity: a novel sign of pancreatic tumor aggressiveness. Clin Cancer Res.

[45] Mankoff DA, Dunnwald LK, Gralow JR, Ellis GK, Charlop A, Lawton TJ (2002). Blood flow and metabolism in locally advanced breast cancer: relationship to response to therapy. J Nucl Med.

[46] Zhao K, Wang C, Mao Q, Shang D, Huang Y, Ma L (2020). The flow-metabolism ratio might predict treatment response and survival in patients with locally advanced esophageal squamous cell carcinoma. EJNMMI Res.

[47] Paschalis A, Sheehan B, Riisnaes R, Rodrigues DN, Gurel B, Bertan C (2019). Prostate-specific membrane antigen heterogeneity and DNA repair defects in prostate cancer. Eur Urol.

[48] Palard-Novello X, Blin AL, Bourhis D, rin E, Salaun PY, Devillers A (2018). Comparison of choline influx from dynamic (18)F-Choline PET/CT and clinicopathological parameters in prostate cancer initial assessment. Ann Nucl Med.

[49] Schaefferkoetter JD, Wang Z, Stephenson MC, Roy S, Conti M, Eriksson L, et al. Quantitative 18F-fluorocholine positron emission tomography for prostate cancer: correlation between kinetic parameters and Gleason scoring. EJNMMI research. 2017;7(1). 10.1186/s13550-017-0269-0 PMC5360745 28324340

[50] Nielsen MM, Tolbod LP, Borre M, Hoyer S, Harms HJ, Sorensen J, et al. The relationship between tumor aggressiveness and cholinergic PET imaging in prostate cancer tissue. A proof-of-concept study. American journal of nuclear medicine and molecular imaging. 2019;9(3):185–92. PMC6627478 31328024

[51] Regula N, Honarvar H, Lubberink M, Jorulf H, Ladjevardi S, Haggman M (2020). Carbon flux as a measure of prostate cancer aggressiveness: [(11)C]-acetate PET/CT. Int J Med Sci.

